# Massive pheochromocytoma

**DOI:** 10.1002/ccr3.3124

**Published:** 2020-07-15

**Authors:** Terumitsu Anai, Kosuke Oka, Yuya Yokota, Yoshito Nishimura, Hideharu Hagiya, Fumio Otsuka

**Affiliations:** ^1^ Department of General Medicine Okayama University Graduate School of Medicine, Dentistry and Pharmaceutical Sciences Okayama Japan

**Keywords:** ^123^I‐metaiodobenzylguanidine scintigraphy, pheochromocytoma, primary care

## Abstract

Complications of pheochromocytoma, such as hypertensive emergency, can be critical. Clinicians should recognize that pheochromocytoma is not uncommon in patients with large adrenal tumors, and screening should be undertaken prior to any intervention.

## Graphical abstract

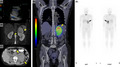

## 1 CASE

**Figure 1:**
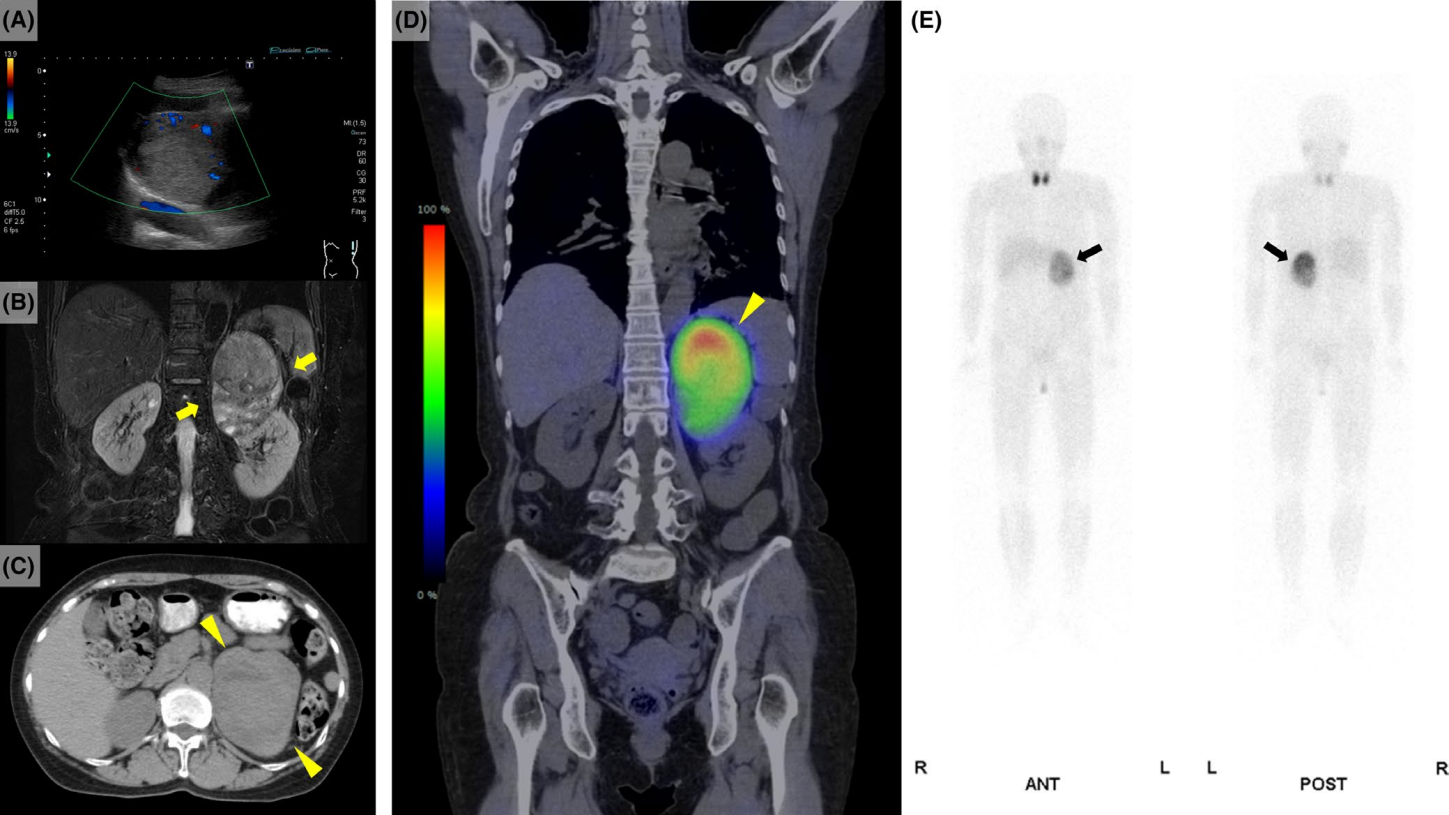
A, Abdominal ultrasonography showing a 11.8 × 7.8 cm massive hypovascular heterogeneous mass with cystic lesions in the left adrenal gland. B, Abdominal noncontrast computed tomography revealing a 10‐cm mass in the left adrenal gland (arrowheads). C, Abdominal fat‐suppression T2‐weighted magnetic resonance imaging showing a well‐circumscribed tumor suggestive of pheochromocytoma. No infiltration of the surrounding tissue is noted. D, E, ^123^I‐metaiodobenzylguanidine scintigraphy revealing intense uptake in the left adrenal tumor

A 63‐year‐old woman without any medical history was referred to our hospital by a primary care doctor due to an adrenal tumor and hypertensive urgency. On admission, her blood pressure was 255/164 mm Hg, but she was otherwise asymptomatic. The catecholamine levels in her plasma (adrenaline, 3.92 ng/mL; noradrenaline, 3.91 ng/mL; dopamine, 0.05 ng/mL) and urine (metanephrine, 29.63 mg/day; normetanephrine, 13.19 mg/day; vanillylmandelic acid, 91.8 mg/day) were markedly elevated. Abdominal ultrasonography revealed a massive hypovascular, heterogeneous mass in the left adrenal gland (Figure 1A). Abdominal computed tomography revealed a 10‐cm mass (Figure 1B), and abdominal fat‐suppression T2‐weighted magnetic resonance imaging showed a well‐circumscribed tumor (Figure 1C). ^123^I‐metaiodobenzylguanidine scintigraphy revealed intense uptake in the left adrenal tumor (Figure 1D, 1E). Based on these findings, the patient was diagnosed with a massive pheochromocytoma. We started administering oral doxazosin and performed laparoscopic left adrenalectomy thereafter. She became normotensive after the surgery.

Pheochromocytoma is a catecholamine‐producing tumor originating in the sympathetic nervous system; it is found in less than 0.2% of patients with hypertension.^1^ Incidence rate of pheochromocytoma was reportedly 0.8 per 100 000 person‐years.^2^ Because there are critical complications such as hypertensive emergency with the disease, early diagnosis of pheochromocytoma is imperative. In particular, pheochromocytoma is not uncommon in patients with large adrenal tumors, and screening for the disease should be undertaken prior to any intervention as of this case.

## CONFLICT OF INTEREST

None declared.

## AUTHOR CONTRIBUTIONS

TA, KO, and YN: wrote the first draft and managed all the submission process. YY, HH, and FO: contributed to the clinical management of the patients and revised the manuscript.
